# 二代测序与FISH检测B细胞淋巴瘤基因重排的一致性比较

**DOI:** 10.3760/cma.j.cn121090-20231225-00340

**Published:** 2024-06

**Authors:** 正 严, 志华 姚, 书娜 姚, 爽 赵, 海英 王, 俊峰 褚, 原林 徐, 九阳 张, 冰 魏, 佳文 郑, 庆欣 夏, 道远 吴, 旭锋 罗, 文平 周, 艳艳 刘

**Affiliations:** 1 郑州大学附属肿瘤医院（河南省肿瘤医院）内科，郑州 450008 Department of Internal Medicine, Affiliated Cancer Hospital of Zhengzhou University（Henan Cancer Hospital）, Zhengzhou 450008, China; 2 郑州大学附属肿瘤医院（河南省肿瘤医院）分子病理科，郑州 450008 Department of Molecular Pathology, Affiliated Cancer Hospital of Zhengzhou University（Henan Cancer Hospital）, Zhengzhou 450008, China; 3 郑州大学附属肿瘤医院（河南省肿瘤医院）病理科，郑州 450008 Department of Pathology, Affiliated Cancer Hospital of Zhengzhou University（Henan Cancer Hospital）, Zhengzhou 450008, China; 4 郑州大学附属肿瘤医院（河南省肿瘤医院）临床研究管理部，郑州 450008 Department of Clinical Research Management, Affiliated Cancer Hospital of Zhengzhou University（Henan Cancer Hospital）, Zhengzhou 450008, China

**Keywords:** 淋巴瘤，B细胞, 原位杂交，荧光, 二代测序, 基因重排, 一致性, Lymphoma, B-Cell, In situ hybridization, fluorescence, Next generation sequencing, Gene rearrangement, Consistency

## Abstract

**目的:**

比较基于二代测序（NGS）的淋巴瘤多基因检测包（panel）与FISH检测B细胞淋巴瘤基因重排的一致性。

**方法:**

收集2019年1月到2023年5月河南省肿瘤医院对489份石蜡包埋的B细胞淋巴瘤组织进行淋巴瘤相关的413个基因靶向捕获测序后发现的融合基因，与同步采用FISH检测BCL2、BCL6、MYC和CCND1 4种断裂/融合基因的结果进行比较。两种方法对同一个样本的检测结果均为阳性或阴性为一致。同时分析NGS中融合突变丰度与FISH中阳性细胞比率的关系。

**结果:**

Kappa一致性分析显示NGS和FISH在检测4种B细胞淋巴瘤相关基因重排上有较高的一致性（*P*值均<0.001），但在阳性个体检出率上4种基因有所不同，NGS对BCL2重排的检出率高于FISH，对BCL6和MYC重排的检出率低于FISH，对CCND1重排的检出率与FISH相同。NGS中融合突变丰度与FISH中阳性细胞比率无相关性。

**结论:**

NGS与FISH检测B细胞淋巴瘤基因重排总体上具有较好一致性。在检测BCL2重排上，NGS优于FISH；在检测MYC重排上，NGS劣于FISH；在检测CCND1重排上二者相当。

## Abstract (en)

**Objective:**

To compare the consistency of lymphoma multigene detection panels based on next-generation sequencing（NGS）with FISH detection of B-cell lymphoma gene rearrangement.

**Methods:**

From January 2019 to May 2023, fusion genes detected by lymphoma-related 413 genes that targeted capture sequencing of 489 B-cell lymphoma tissues embedded in paraffin were collected from Henan Cancer Hospital, and the results were compared with simultaneous FISH detection of four break/fusion genes: BCL2, BCL6, MYC, and CCND1. Consistency was defined as both methods yielding positive or negative results for the same sample. The relationship between fusion mutation abundance in NGS and the positivity rate of cells in FISH was also analyzed.

**Results:**

Kappa consistency analysis revealed high consistency between NGS and FISH in detecting the four B-cell lymphoma-related gene rearrangement（*P*<0.001 for all）; however, the detection rates of positive individuals differed for the four genes. Compared with FISH, NGS demonstrated a higher detection rate for BCL2 rearrangement, a lower detection rate for BCL6 and MYC rearrangement, and a similar detection rate for CCND1 rearrangement. No correlation was found between fusion mutation abundance in NGS and the positivity rate of cells in FISH.

**Conclusion:**

NGS and FISH detection of B-cell lymphoma gene rearrangement demonstrate overall good consistency. NGS is superior to FISH in detecting BCL2 rearrangement, inferior in detecting MYC rearrangement, and comparable in detecting CCND1 rearrangement.

B细胞淋巴瘤相关基因重排的检测是临床常用的分子诊断手段。FISH是检测基因重排的金标准，但存在缺点：依赖被检组织形态结构的完整性，对人工依赖性高，1次只能检测1种重排，检测少见的断裂位点、多倍体或复杂荧光信号时存在困难，断裂探针不能识别伴侣基因，而融合探针只适用于检测伴侣基因和断裂位点已知的重排[1]。此外，不同厂家生产的FISH探针检测的结果存在较高的不一致性[2]，且不同实验室判读阳性的标准存在差异。近年来，二代测序（NGS）被广泛地应用于临床，淋巴瘤相关多基因检测包（panel）在检测基因突变的同时可以检测基因重排，并能够识别断裂位点和伴侣基因。随着NGS的大规模应用，比较NGS和FISH检测淋巴瘤基因重排的优劣势在必行。本研究对这两种方法检测4种常见基因（BCL2、BCL6、MYC和CCND1）重排的结果进行了比较。

## 病例与方法

1. 病例资料：河南省肿瘤医院内科在2019年初启动了一项“淋巴瘤驱动基因突变的意义和靶向治疗研究”的前瞻性研究（NCT04263935），采用淋巴瘤相关多基因检测包对收治的淋巴瘤患者的石蜡包埋组织进行NGS，该基因检测包在检测413个淋巴瘤相关的基因突变的同时能检测淋巴瘤有关的基因重排。部分患者同时采用了FISH检测BCL2、BCL6、MYC和CCND1中的一种或多种基因的重排。从2019年1月1日至2023年5月27日，共495例B细胞淋巴瘤患者进行了NGS，这些患者中同时用FISH检测BCL2 179例，检测BCL6 143例，检测MYC 163例，检测CCND1 21例，其中6例的测序与FISH所用标本不是同一时间或同一部位的活检组织，不纳入比较。在剩余的489例测序的患者中，弥漫大B细胞淋巴瘤（DLBCL）348例，滤泡性淋巴瘤（FL）58例，儿童FL 2例，套细胞淋巴瘤29例，边缘区淋巴瘤20例，伯基特淋巴瘤19例，华氏巨球蛋白血症5例，慢性淋巴细胞白血病/小淋巴细胞淋巴瘤4例，未能分类B细胞淋巴瘤2例，B淋巴母细胞淋巴瘤1例，原发纵隔大B细胞淋巴瘤1例。同时用FISH检测BCL2 174例，BCL6 138例，MYC 158例，CCND1 19例。

2. 测序方法：使用DNA提取试剂盒（德国Qiagen公司）从肿瘤组织中提取基因组DNA，取400 ng DNA使用超声波破碎仪进行片段化；随后按照建库说明书制备测序文库。制备好的DNA文库与特殊定制的探针进行杂交以捕获目标序列。特殊定制的探针全面覆盖WHO、中国临床肿瘤学会（CSCO）和NCCN等指南中涉及淋巴瘤患者辅助诊断、预后评估及靶向用药相关的基因，检测的基因变异类型包括点突变、小片段插入缺失和特定融合基因（用药及预后相关重点基因的全部蛋白质编码序列区及部分非编码区域）。检测融合基因的探针根据文献报道的相关靶基因的热点断裂位置设计，包含热点的内含子以及基因间区（表1）[3]–[5]。最终的文库使用Qubit™高敏试剂盒（美国赛默飞世尔科技公司）进行定量评估，同时使用高敏DNA分析试剂盒（美国安捷伦公司）进行质量评估。捕获的DNA片段通过MGISEQ-2000测序仪（中国深圳华大智造科技有限公司）进行测序。数据信息分析之前首先保证质控参数的合格，质控参数主要包括下机数据量、靶向捕获效率、平均测序深度、100×覆盖度等。质控合格后的数据使用bwa-mem工具（0.7.15版本）与参考基因组（hg37）进行基因组比对[6]，随后使用VarDict（1.4.6版本）及Varscan（2.4.2版本）对点突变、小片段插入缺失进行变异检测[7]–[8]，同时被两种算法鉴定到的变异使用SnpEff（4.3版本）进行注释[9]。变异结果的删选标准为：检测深度≥500×，变异丰度≥2％，此外，在1000G、ESP、ExAC等数据库中的人群频率均需<5％。使用FACTERA进行基因融合的检测[10]，选择Soft-clipped reads>10条的断裂融合位置，随后利用IGV软件人工判断基因融合的可信度及其融合形式。

**表1:** 融合基因检测的范围和靶向捕获区域

目标基因	捕获区域	基因坐标
BCL2	全CDS，3′UTR（3号外显子下游30 kb区域）	Chr18：60795829-60796006、60985154-60986638
BCL6	全CDS，1号内含子	Chr3：187439759-187440433、187442704-187442880、187443264-187443430、187444489-187444702、187446124-187446371、187446814-187447825、187449464-187449749、187451299-187451513
MYC	全CDS，1号内含子	Chr8：128746807-128748893、128748902-128751289、128752612-128753235
CCND1	全CDS，选择性区域	Chr11：69456056-69456308、69457776-69458045、69458571-69458782、69462726-69462943、69465861-69465967、69465991-69466066

**注** CDS：蛋白质编码区；UTR：非翻译区

3. FISH：FISH检测所使用的融合或断裂探针为广州安必平医药科技股份有限公司生产。检测方法参考文献[11]。BCL2、MYC和CCND1重排的检测，部分使用了融合探针。BCL6重排的检测全部使用了断裂探针。阳性判读阈值如下：BCL2断裂15％、BCL2::IgH 5％、BCL6断裂15％、MYC断裂10％、MYC::IgH 5％、CCND1断裂15％、CCND1::IgH 8％。

4. 比较及分析方法：所有经过测序的病例均有BCL2、BCL6、MYC和CCND1的重排信息，检出重排者视为阳性，未检出重排视为阴性。部分病例进行过一种或多种基因的FISH检测。在比较一致性时，NGS和FISH的结果同时为阴性或阳性，视为一致；一种检测结果为阴性而另一种检测结果为阳性，视为不一致。在分析重排基因的伴侣基因时纳入所有接受测序的B细胞淋巴瘤患者。

5. 统计学处理：NGS和FISH检测基因重排的一致性采用Kappa检验。采用率描述阳性事件的发生频次，组间率的比较采用Fisher精确概率法。NGS中的融合突变丰度和FISH中的阳性细胞比率分析采用Pearson相关分析。所有的统计分析采用SPSS 25.0完成。*P*<0.05为差异具有统计学意义。

## 结果

1. NGS和FISH检出的B细胞淋巴瘤相关基因重排的比较：如表2所示，两种检测方法的一致性较高，对四个基因重排的检测一致性均达到90％以上（*P*值均<0.001）。由于临床更加关注阳性检出率，经比较，NGS对BCL2重排的阳性检出率高于FISH（93.5％对80.6％，*P*＝0.250），在8例检测结果不一致的患者中NGS检测阴性而FISH检测阳性（NGS^−^/FISH^+^）2例，NGS^+^/FISH^−^ 6例，这6例的伴侣基因为IgH；NGS对BCL6重排的阳性检出率低于FISH（68.8％对90.6％，*P*＝0.060），13例检测结果不一致的患者中NGS^−^/FISH^+^ 10例，NGS^+^/FISH^−^ 3例，这3例均为非重现性融合（BCL6::非Ig）；NGS对MYC重排的阳性检出率也低于FISH（67.7％对100％，*P*<0.001），10例不一致的均是NGS^−^/FISH^+^；两者对CCND1重排的检出率均达到100％。

**表2:** 二代测序（NGS）和FISH检测BCL2、BCL6、MYC和CCND1基因重排的一致性分析

基因	例数	检测结果[例（%）]		两种方法检出阳性总例数	NGS检出阳性例数	FISH检出阳性例数	Kappa统计值	*P*值
		一致	不一致					
BCL2	174	166（95.4）	8（4.6）	31	29	25	0.825	<0.001
BCL6	138	125（90.6）	13（8.4）	32	22	29	0.689	<0.001
MYC	158	148（93.7）	10（6.3）	31	21	31	0.771	<0.001
CCND1	19	19（100）	0（0）	11	11	11	1.000	<0.001

2. NGS中融合突变丰度和FISH中阳性细胞比率之间的关系：NGS检出的融合基因的突变丰度反映了发生融合的基因拷贝数，也一定程度上反映了发生融合突变细胞的比率。于是我们对NGS中融合突变丰度和FISH中阳性细胞比率进行了Pearson相关分析，但结果显示两者间并无相关性（BCL2、BCL6和CCND1相关分析的*r*值分别为0.150、0.153和0.234，*P*值分别为0.237、0.338和0.085）。提示可能在肿瘤细胞内，不仅发生了基因组结构异常（基因融合），同时基因组数目也发生改变，表明肿瘤细胞的基因组变异的多样性。

3. NGS检出的B细胞淋巴瘤相关的融合基因：在接受NGS的495例B细胞淋巴瘤患者中，254例未检出任何基因重排，另外的241例患者中共检出273个重排基因。涉及BCL2的重排64个，BCL6的重排97个，MYC的重排37个，CCND1的重排26个。如图1所示，BCL2、MYC和CCND1的伴侣基因高度集中，绝大部分是IgH，偶见其他伴侣基因且都是非重现性重排；而BCL6的伴侣基因具有高度多样性，约一半为IgH，其他的伴侣基因中除了IgK和IgL之外几乎都是非重现性重排。

**图1:**
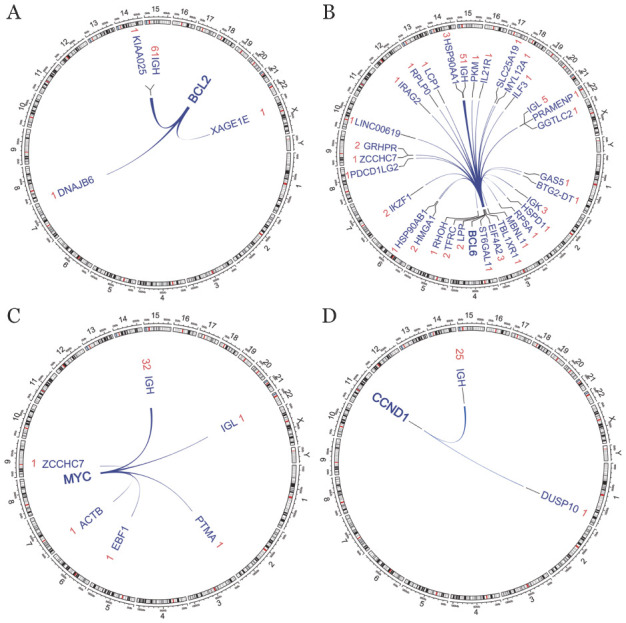
环图展示二代测序检出的基因重排 **注** A、B、C和D分别展示BCL2、BCL6、MYC和CCND1相关的重排；弧线的两端指向重排的两个伴侣基因，弧线的宽度代表该重排发生的频率高低，基因名称旁边红色的数字表示该重排的检出例数，环外的数字或字母表示染色体编号

为了进一步检验NGS检测基因重排的可靠性，我们从有剩余组织的病例中各选取1例NGS检出BCL2（此例NGS^+^/FISH^−^）、MYC和CCND1重排的样本，用PCR扩增后进行Sanger测序。如图2所示，NGS可以在单个碱基分辨率上显示融合位点和伴侣基因的序列，并且与Sanger测序获得的碱基序列完全一致。

**图2:**
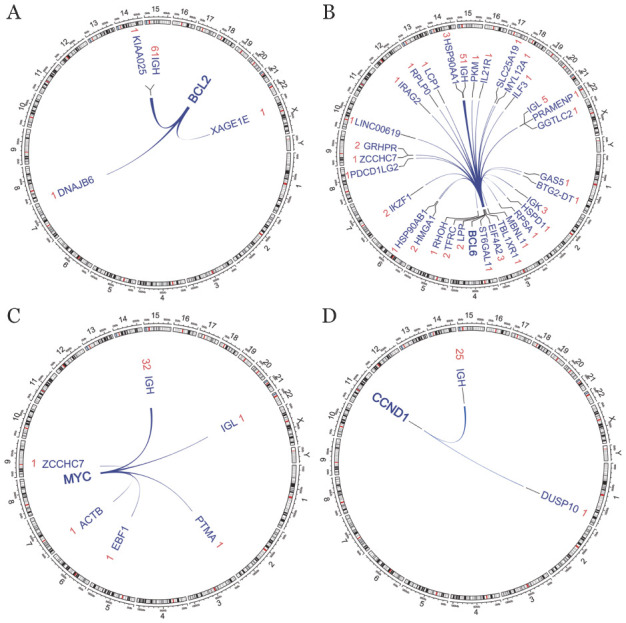
代表性二代测序（NGS）和Sanger测序获得的基因重排序列的对照 **注** A、C和E分别是NGS测得的BCL2::IgH、MYC::IgH和CCND1::IgH重排序列；B、D和F分别是Sanger测序获得的BCL2::IgH、MYC::IgH和CCND1::IgH重排序列；NGS在单个碱基分辨率上显示融合位点和伴侣基因的序列，并且与Sanger测序获得的碱基序列完全一致。黄色箭头指向融合位置

## 讨论

本研究在较大样本的B细胞淋巴瘤中比较了NGS和FISH检测基因重排的一致性。总体来看两种方法检测BCL2、BCL6、MYC和CCND1 4种常见基因重排的一致性较好。在既往的几项国外的研究中，NGS和FISH的结果都不完全一致[2],[4],[12]。我们结合其他临床信息对不一致的个案做了进一步的分析和推断，供读者参考。在BCL2不一致的8例中，6例NGS^+^/FISH^−^，其中5例病理诊断为FL，且其中1例经过Sanger测序证明存在BCL2::IgH，因此这5例的FISH结果很可能是假阴性（FISH漏检），另外1例无法推断；2例NGS^−^/FISH^+^，其中1例病理诊断为FL，因此NGS的结果可能是假阴性，另外1例无法推断。在BCL6不一致的13例中，3例NGS^+^/FISH^−^，无法推断；10例NGS^−^/FISH^+^，其中6例FISH阳性细胞比率处在临界或接近临界状态（15％～20％），FISH有假阳性的可能，1例FISH为不典型阳性，也有假阳性的可能，2例FISH阳性细胞比率高（>60％），NGS有假阴性的可能，另外1例无法推断。MYC不一致的10例均为NGS^−^/FISH^+^，其中2例病理诊断为伯基特淋巴瘤，且FISH阳性细胞比率高（>60％），因此这2例的NGS很可能是假阴性（NGS漏检）；2例其他类型B细胞淋巴瘤的FISH阳性细胞比率高（>60％），NGS有假阴性的可能；另外6例无法推断。这些不一致的问题有待进一步研究，结合上述推断，我们认为在检测BCL2重排上NGS优于FISH，在检测MYC重排上NGS劣于FISH，在检测BCL6上仍无法下结论。

从重排的伴侣基因看，BCL2的伴侣基因相对集中，绝大部分是IgH，而BCL6的伴侣基因呈现高度的多样性，与国外在DLBCL上的研究结果一致[13]。此前国外的研究中，部分MYC重排的伴侣是非Ig基因，NGS检测MYC::Ig具有高敏感性，而检出MYC::非Ig的敏感性不如FISH[12]。本研究中只检出了很少的MYC::非Ig，推测NGS漏检（NGS^−^/FISH^+^）的很可能是MYC::非Ig。大部分MYC::非Ig的断裂点发生在MYC的基因簇区以外[2]，这可能是NGS漏检的原因。

尽管NGS中融合突变频率和FISH中阳性细胞比率都代表发生融合变异的基因拷贝数占该基因座位上全部基因拷贝数的比率，但两者在计算时分母所代表的细胞群大不相同。NGS是从整个切片中提取DNA，除了肿瘤细胞外，还包括大量非肿瘤细胞的DNA，且非肿瘤细胞的构成比变异性强；而FISH是选取切片中最具有代表性的区域（一般是肿瘤细胞密集区）进行细胞计数，受非肿瘤细胞的影响小。这可能是NGS中融合突变频率与FISH中阳性细胞比率之间相关性差的主要原因。

虽然NGS检测部分B细胞淋巴瘤相关的基因重排不如FISH敏感，但随着NGS技术的进步和优化，我们认为其在将来有取代FISH的可能。
